# Antibacterial Activity and Mode of Action of Dihydromyricetin from *Ampelopsis grossedentata* Leaves against Food-Borne Bacteria

**DOI:** 10.3390/molecules24152831

**Published:** 2019-08-03

**Authors:** Xiao-Nian Xiao, Fan Wang, Yi-Ting Yuan, Jing Liu, Yue-Zhen Liu, Xing Yi

**Affiliations:** 1 OAI Sino-German United Research Institute, Nanchang University, Nanchang 330047, Jiangxi Province, China; 2 Sino-German Food Engineering Center, Nanchang University, Nanchang 330047, Jiangxi Province, China

**Keywords:** dihydromyricetin, food-borne bacteria, antibacterial activity, mechanism

## Abstract

Dihydromyricetin (DMY) has recently attracted increased interest due to its considerable health-promoting activities but there are few reports on its antibacterial activity and mechanism. In this paper, the activity and mechanisms of DMY from *Ampelopsis grossedentata* leaves against food-borne bacteria are investigated. Moreover, the effects of pH, thermal-processing, and metal ions on the antibacterial activity of DMY are also evaluated. The results show that DMY exhibits ideal antibacterial activity on five types of food-borne bacteria (*Staphylococcus aureus, Bacillus subtilis, Escherichia coli, Salmonella paratyphi,* and *Pseudomonas aeruginosa*). The activities of DMY against bacteria are extremely sensitive to pH, thermal-processing, and metal ions. The morphology of the tested bacteria is changed and damaged more seriously with the exposure time of DMY. Furthermore, the results of the oxidative respiratory metabolism assay and the integrity of the cell membrane and wall tests revealed that the death of bacteria caused by DMY might be due to lysis of the cell wall, leakage of intracellular ingredients, and inhibition of the tricarboxylic acid cycle (TCA) pathway.

## 1. Introduction

Food spoilage, which leads to huge economic losses, shelf-life reduction, and food-borne diseases in the food industry every year, is the most prominent problem in food quality and safety. The essence of food spoilage is attributed to a series of spoilage substances produced by the microbial decomposition of some nutrients during their growth and metabolism. Therefore, the microorganisms are mainly responsible for this problem [1]. In recent years, the synthetic preservatives have been widely applied in food due to their low cost and ideal antibacterial capacity. However, synthetic preservatives have not been widely accepted by consumers due to their assumed adverse effects on health [2]. Besides, the addition of these preservatives is also restricted because of their toxicity. In consideration of this situation, many studies have explored the ability of natural antimicrobial components to replace normal synthetic preservatives, including phytochemicals (i.e., flavonoids [3], polyphenols, terpenoids [4], and isothiocyanates [5]), essential oils [6], antimicrobial enzymes (i.e., lysozyme [7] and lactoperoxidase [8]), and antimicrobial peptides [9]. Until recently, the proposed antibacterial mechanisms of these natural preservatives could be divided into the following types: changes in the membrane permeability, alterations in cytoplasmic membrane function, energy metabolism inhibition, reduction in cell attachment and biofilm formation, nucleic acid synthesis inhibition, inhibition of the porin on the cell membrane, attenuation of the pathogenicity, and cytoplasmic membrane damage [10].

Dihydromyricetin (DMY), also known as ampelopsin, belongs to the flavonoid family and is a major secondary metabolite of *Ampelopsis grossedentata*. Its leaves are extremely rich in DMY, and it is used as the preferred material for large-scale DMY extraction [11]. Recent studies have demonstrated that DMY has many different biological activities, including anti-inflammatory [12], anticancer [13], antioxidant [14], neuroprotective [15], and metabolism-regulatory (lipid and glucose) activities [16], and thus DMY is widely applied in the food, pharmaceutical, and cosmetics industries. Many studies have also confirmed that DMY has ideal antimicrobial activity against *Staphylococcus aureus* [17], *Escherichia coli* [18], *Streptococcus* [19], *Aspergillus niger* [20], and *Pseudomonas aeruginosa* [21]. However, there is still a lack of research about the antibacterial activity and mechanisms of DMY against some food-borne bacteria. In addition, DMY is extremely unstable due to its five phenolic hydroxyl groups, especially ortho-trihydroxyl groups in the B ring. Some food storage and processing conditions, such as pH, temperature, and metal ions, can cause changes in the DMY structure, thus affecting its biological activities.

The aim of this study is to evaluate the antibacterial activity of DMY against five food-borne bacteria, investigate the effects of pH, thermal-processing, and metal ions on the antibacterial activity of DMY and disclose its mechanisms of antibacterial action by morphology observation, integrity of the cell membrane and wall, permeability of the cell membrane, and oxidative respiratory metabolism characteristics.

## 2. Results and Discussion

### 2.1. Antibacterial Activity of Dihydromyricetin (DMY)

**Figure 1:**
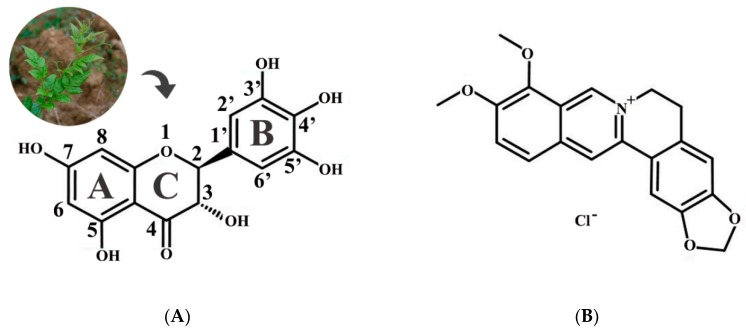
Chemical structures of DMY (**A**) and Berberine hydrochloride (BH) (**B**).

The antibacterial activity of DMY was preliminary investigated by the disc-diffusion method using five food-borne microorganisms including two strains of Gram-positive bacteria (*S. aureus* and *B. subtilis*) and three strains of Gram-negative bacteria (*E. coli*, *S. paratyphi,* and *P. aeruginosa*), and revealed by the diameters of the inhibition zone (DIZs) observed on the agar plate (Table 1). The antibacterial ability was classified according to the DIZ as follows: not sensitive (DIZ < 8.0 mm), moderately sensitive (8.0 < DIZ < 14.0 mm), sensitive (14.0 < DIZ < 20.0 mm), and extremely sensitive (DIZ > 20.0 mm) [22]. The results showed that DMY exhibited moderate activity against both Gram-positive bacteria and Gram-negative bacteria even at a low concentration (3.78 mg/mL), with the maximum DIZ value being for *S. aureus* (10.6 ± 0.5 mm), followed by *S. paratyphi* (10.5 ± 0.3 mm), *P. aeruginosa* (10.1 ± 0.3 mm), *E. coli* (9.8 ± 0.3 mm), and *B. subtilis* (8.1 ± 0.2 mm). When the DMY concentration was 11.34 mg/mL, most of the tested bacteria, such as *S. aureus*, *E. coli,* and *P. aeruginosa*, were sensitive to DMY with DIZ values ranging from 15 to 18 mm. Therefore, DMY had certain antibacterial activity against all of the tested bacteria. Some previous studies confirmed that hydroxylation at position 5 and 7 plays an important role in the antibacterial activity of flavonols against bacteria, and hydroxylation on the B and C rings can also improve this ability [23,24] (Figure 1A). Moreover, Wu et al. [25] reported the hydroxylation at position 3 and carbonyl oxygen at position 4 on the C ring are not necessary for the antibacterial activity of DMY against *S. aureous*. Accordingly, hydroxylation at positions 5, 7, 3′, 4′, and 5′ may enable DMY to possess wide antibacterial activity, but the exact positions of antibacterial action require further study.

Berberine hydrochloride (BH) (Figure 1B), which is a typical bacterial inhibitor with a broad antibacterial spectrum, was used for comparison to evaluate the antibacterial ability of DMY. The minimum inhibitory concentration (MIC) and minimum bactericidal concentration (MBC) of DMY and BH for five food-borne microorganisms were determined and are shown in Table 1. The MICs and MBCs of DMY were in the ranges of 0.3125–2.5 mg/mL and 2.5–10 mg/mL, respectively. DMY exhibited the same inhibitory and bactericidal activity as BH against Gram-positive bacteria, and Gram-negative bacteria were more sensitive to DMY than BH. Generally, Gram-negative bacteria should have been less sensitive to the bacterial inhibitor than Gram-positive bacteria due to the more complex cell surface characteristics and structures, however, this did not occur in DMY [26]. This might be because the presence of structural lipopolysaccharide molecules in the outer membrane provided a hydrophilic surface for Gram-negative bacteria and DMY was able to penetrate into the target cell membrane without blockage due to its highly hydrophilic character [27]. Accordingly, Gram-negative bacteria were not resistant to DMY. These results indicated the DMY has noticeable antibacterial activity and could be applied as a potential natural bacterial inhibitor with a broad antibacterial spectrum to replace some synthetic preservatives in food.

### 2.2. Effect of Calcium Ions, Thermal Processing, and pH on the Antibacterial Activity of DMY

**Figure 2:**
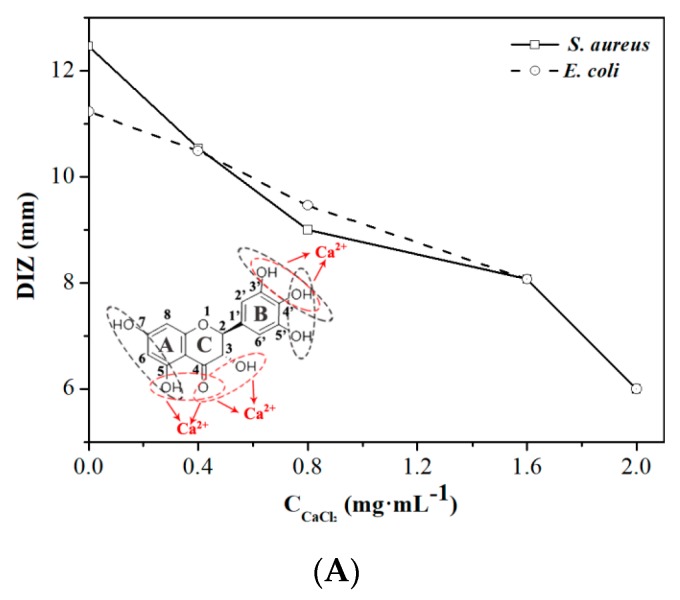

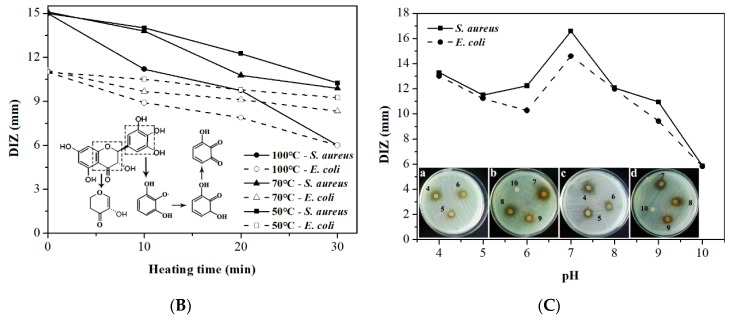
The effects of (**A**) Ca^2+^, (**B**) thermal processing, and (**C**) pH on the activities of DMY against *E. coli* and *S. aureus*.

Metal ions (i.e., Na^+^, Ca^2+^, Fe^3+^, and Al^3+^) exist in many kinds of food material such as fruits, vegetables, condiments, dairies, and additives, and they are essential for food consumption and conservation [28]. In order to investigate the effects of metal ions on the antibacterial activity of DMY, Ca^2+^, which widely exists in food, was applied to treat DMY, and DIZ was used to evaluate its antibacterial activity. As shown in Figure 2A, the antibacterial activities of DMY against *E. coli* and *S. aureus* both decreased with an increase in the concentration of Ca^2+^. According to previous studies, metal ions have no effect on the stability of DMY [29], but they will interact with DMY to form chelates. Normally, there are three domains that can interact with metal ions in each flavonoid molecule (red circles) including the 3′,4′-dihydroxy group in the B ring and the 3-hydroxy or 5-hydroxy and the 4-carbonyl groups in the C ring [30]. Most of these sites also play important roles in the activity of DMY against bacteria (black circles) (Figure 2A inset). Therefore, the formation of DMY-Ca^2+^ chelate might be put an obstacle to the interaction between DMY and bacteria and thus may decrease the antibacterial activity of DMY.

Heating (i.e., boiling, roasting, drying, frying, sterilization, and baking), which have been widely used to improve the quality, safety, and shelf-life of foods, are commonly used thermal processing techniques in the food industry [31]. Thermal processing has the potential to alter the activity of natural bacterial inhibitors, and the degree of such impact mainly depends on the heating conditions, such as temperature and time. Therefore, the antibacterial activity of DMY treated with different heating conditions was investigated, as shown in Figure 1B. The results showed that the activity of DMY against *E. coli* and *S. aureus* significantly decreased with heating time and the increase of temperature, and was completely lost after being heated at 100 °C for 30 min. This might be due to the thermal instability of DMY in solution. Xin et al. [32] confirmed decomposition of DMY will not occur below 240 °C according to the results of thermal gravimetric analysis, but DMY can be irreversibly oxidized to quinone compounds or myricetin above 30 °C due to its ortho-trihydroxyl groups at position 3′, 4′, and 5′ and active α-hydrogen of carbonyl group [33,34] (Figure 2B inset). Therefore, the changes of DMY molecular structure caused by oxidation under thermal-processing were responsible for the decrease of antibacterial activity.

The antibacterial activities of DMY against *E. coli* and *S. aureus* at different pH levels are shown in Figure 2C. The results indicate that pH played a significant role in the antibacterial activity of DMY. The lower pH (<7) was beneficial for the antibacterial activity of DMY. Epand et al. [35] reported that cationic charges can help some bacterial inhibitors to bind with the anionic lipid components of the bacterial cell membrane. In this study, the protonation of five phenolic hydroxyl groups on the DMY molecules in acid medium might play an important role in this binding process by enhancing the antibacterial activity. In addition, Xiang et al. [36] confirmed that DMY can remain stable under the pH range of 1.0~5.0. These factors might contribute to the high and stable antibacterial activity of DMY under acidic conditions. Noticeably, the apparent color of the DMY solution varied from yellow to brown in the pH range of 8.0 to 10.0 (Figure 2a–d). According to previous studies [29], it can be inferred from this phenomenon that the structure of the DMY molecule might have changed and dramatic degradation may have occurred. Furthermore, Xin et al. [32] reported that the 7-hydroxyl group and some of the ortho-trihydroxyl groups of DMY can be ionized under neutral or alkaline conditions. Accordingly, degradation and ionization led to the extreme instability of DMY under neutral or alkaline conditions and thus the activities of DMY against both *E. coli* and *S. aureus* rapidly decreased under the pH range of 8.0–10.0.

### 2.3. Morphology of Bacteria Treated with DMY

**Figure 3:**
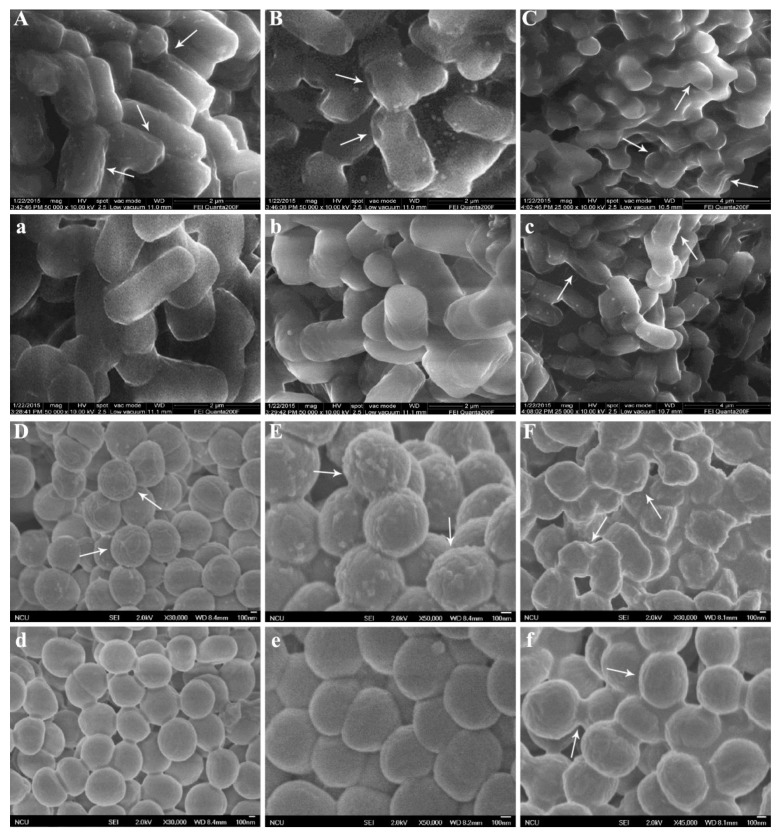
Morphology of bacteria treated (uppercase letters) and untreated (lowercase letters) with DMY. (**A** and **a**): *E. coli* for 4 h, (**B** and **b**): *E. coli* for 14 h, (**C** and **c**): *E. coli* for 24 h, (**D** and **d**): *S. aureus* for 4 h, (**E** and **e**): *S. aureus* for 14 h, (**F** and **f**): *S. aureus* for 24 h.

In order to further explore the mechanism of DMY against bacteria, scanning electron microscopy (SEM) was used to observe the cell morphological changes and membrane damage. The morphologies of *E. coli* and *S. aureus* treated and untreated with DMY are shown in Figure 2. As is presented in Figure 3a,d, untreated *E. coli* exhibited regular, rod-shaped, striated cells and an intact surface, and untreated *S. aureus* remained spherical, regular, and intact, and the cell surfaces were smooth. Although the morphology of treated *E. coli* and *S. aureus* did not change significantly, a few irregular structures appeared on the cell surfaces after 4 h (Figure 3A,D). This suggests that the permeability of the bacterial cell membranes might have increased, but these bacteria were not killed [37]. After being treated with DMY for 14 h (Figure 3B,E), the cell membranes were significantly shriveled, with some little holes on the cell surfaces compared with untreated bacteria (Figure 3b,e). When exposed to DMY for 24 h, the cell surfaces of both *E. coli* and *S. aureus* were severely irregular, coarse, and shriveled, and aggregations and adhesions also appeared between bacteria (Figure 3C,F). Moreover, their sizes and distributions were not uniform. El-Maati et al. [38] and Anwar et al. [39] also reported the same impact of natural antibacterial agents, such as phenolic compounds and flavonoids, on the morphologies of *E. coli* and *S. aureus*. However, only a few irregular structures appeared on the cell surfaces of untreated *E. coli* and *S. aureus* after 24 h (Figure 3c,f). These phenomena might be attributed to the destruction of the cell membranes and the losses of intracellular materials caused by DMY [27]. Furthermore, the mechanisms of DMY against these bacteria need to be confirmed from more perspectives.

### 2.4. Integrity of Cell Membranes and Walls

**Figure 4:**
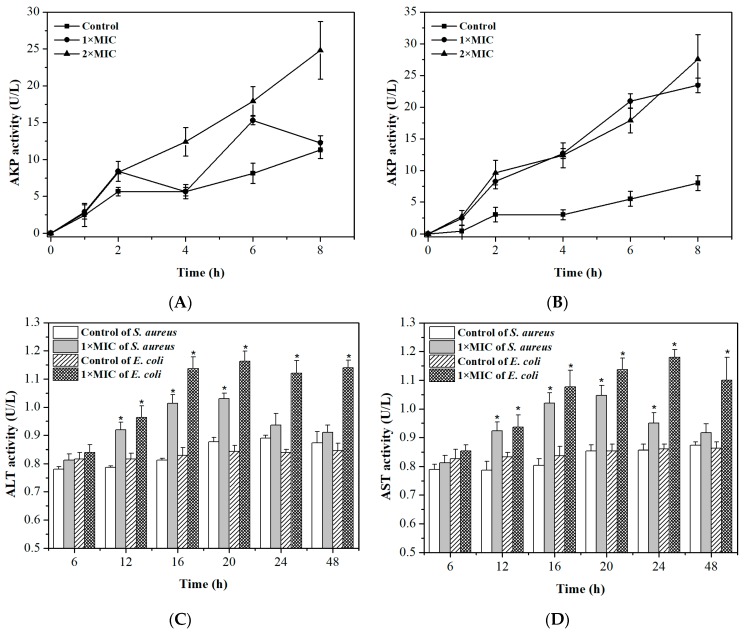
Alkaline phosphatase (AKP) activity of *S. aureus* (**A**) and *E. coli* (**B**) before and after DMY treatment. (**C**) Alanine transaminase (ALT) and (**D**) aspartate transaminase (AST) activity of *S. aureus* and *E. coli* before and after DMY treatment. * *p* < 0.05 versus the control group.

Alkaline phosphatase (AKP), which is a kind of intracellular enzyme, is located between the cell wall and membrane [40]. Alanine transaminase (ALT) and aspartate transaminase (AST) are also intracellular enzymes, but they mainly exist in intracellular fluid [41]. Generally, their activity cannot be detected in the extracellular environment if bacteria possess undamaged cell membranes and walls. The release of AKP, ALT, and AST is shown in Figure 4. As shown in Figure 4A,B, in the absence of DMY, the AKP activity of *S. aureus* and *E. coli* increased to 11.03 and 8.27 U/L respectively, after 8 h. After being treated by DMY for 8 h, the AKP activity of *S. aureus* and *E. coli* increased to 12.41 and 23.43 U/L at MIC and 24.81 and 27.57 U/L at 2 × MIC, respectively. Noticeably, the AKP activity from both bacteria also increased with an increase in the DMY concentration from 1 × MIC to 2 × MIC. Furthermore, it was observed in Figure 4C,D that the ALT and AST activity of *S. aureus* and *E. coli* were significantly higher than that of control groups. Besides, the ALT and AST activity decreased after 20 h, which may have been caused by the low bacterial metabolism activity in the decline period. The release of intracellular constituents demonstrates that DMY might damage the cell wall and membrane, leading to the leakage of AKP, AST, and ALT from cells with a loss of cytoderm integrity, and consequently, leading to bacterial death [42].

### 2.5. Permeability of Cell Membrane

**Figure 5:**
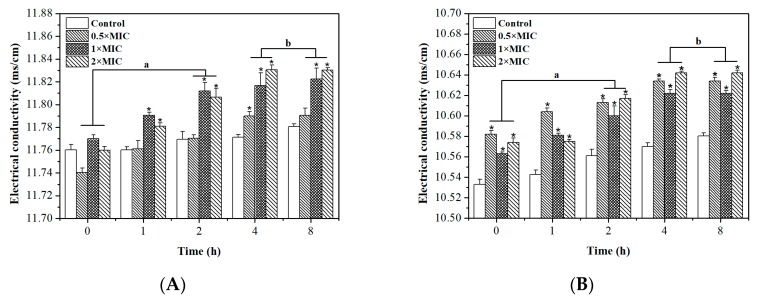
The effects of DMY on the permeability of *S. aureus* (**A**) and *E. coli* (**B**). * *p* < 0.05 versus the control group. ^a^ There was a significant difference between two intervals (*p* < 0.05). ^b^ There was no significant difference between the two intervals (*p* > 0.05).

A permeability barrier is provided by the inner bacterial membrane that allows the passage of small ions which participate in the maintenance of some enzyme activities, keeping the normal metabolism and facilitating cell membrane function [43]. Accordingly, the disruption of cell membrane permeability can cause the leakage of electrolytes and is one of the antibacterial mechanisms [44]. The electrical conductivity was determined to investigate the change in cell membrane permeability. Figure 5 shows that the electrical conductivity of control groups of *S. aureus* and *E. coli* increased from 11.760 ± 0.005 to 11.781 ± 0.003 ms/cm and from 10.533 ± 0.005 to 10.580 ± 0.003 ms/cm respectively, after 8 h. This might be due to the normal lysis and death of the bacteria. The electrical conductivities of *S. aureus* increased to 11.791 ± 0.006, 10.812 ± 0.010, and 11.830 ± 0.002 ms/cm after treatment with 0.5 × MIC, 1 × MIC, and 2 × MIC DMY for 8 h, respectively. In addition, the electrical conductivity of *E. coli* treated with DMY for 8 h also increased to 10.634 ± 0.004, 10.622 ± 0.003, and 10.642 ± 0.003 ms/cm at 0.5 × MIC, 1 × MIC, and 2 × MIC, respectively. Noticeably, the electrical conductivity of *S. aureus* and *E. coli* treated with DMY was significantly higher than that of control groups (*p* < 0.05). These results indicate that DMY could change the permeability of the bacteria membrane, leading to leakage of the intracellular ingredients and cell death [45]. Moreover, the electrical conductivity obtained with 0.5 × MIC, 1 × MIC, and 2 × MIC at intervals of 4 h and 8 h were almost the same (*p* > 0.05) regarding both *S. aureus* and *E. coli*. Thus, it was concluded that 2 h of exposure might be the time that would cause the maximum changes in the cell membrane to occur.

### 2.6. Oxidative Respiratory Metabolism Characteristics

Glucose decomposition of microorganism occurs through three pathways including the hexose monophophate pathway (HMP), the Embden-Meyerhof-Parnas pathway (EMP), and the tricarboxylic acid cycle (TCA) [46]. The three corresponding typical respiratory inhibitors are sodium phosphate tribasic dodecahydrate, iodoacetic acid, and malonic acid. The superposition rates between three typical metabolic inhibitors and the antibacterial agent were determined to analyze the respiration inhibition rates of the bacteria [47]. The inhibitory activities of DMY and the three typical respiratory inhibitors against the respiration rates of *E. coli* and *S. aureus* are shown in Table 2. The inhibition rate of DMY was 20.62% against *S. aureus* and 19.24% against *E. coli*. Malonic acid showed the highest inhibition rate against *S. aureus* and *E. coli* with the I_R_% of 28.26% and 24.18%, respectively. Furthermore, the superposition rate between DMY and each typical respiratory inhibitor was also calculated, and these values are shown in Table 2. The S_R_% values of DMY combined with malonic acid, iodoacetic acid, and sodium phosphate were 24.18%, 8.26%, and 17.26% against *E. coli* respectively, and 28.26%, 7.47%, and 19.18% against *S. aureus*, respectively. The lowest superposition rate represents the highest probability of an inhibiting pathway forming [48]. Therefore, these results imply that the respiratory metabolism of *S. aureus* and *E. coli* was inhibited by DMY through the TCA pathway.

## 3. Materials and Methods

### 3.1. Plant Material

*Ampelopsis Grossedentata* leaves were purchased from Xinglanshan Biotech Co., Ltd. (Jiangxi Province, China) and ground to a fine power in a grinder for extraction.

### 3.2. Microorganisms

*Staphylococcus aureus* ATCC 6538, *Bacillus subtilis* ATCC 6633, *Escherichia coli* ATCC 29194, *Salmonella paratyphi* ATCC 11,511, and *Pseudomonas aeruginosa* ATCC 15,442 were obtained from China Center of Industrial Culture Collection (CICC).

### 3.3. Extraction, Purification, and Determination of DMY

The DMY was extracted from *Ampelopsis grossedentata* leaves using the method established by Gao et al. [49] with some modifications. *Ampelopsis grossedentata* leaves (25 g) were extracted by reflux with 90% ethanol (500 mL) at 80 °C for 2 h and then filtered. The filtrate was concentrated under vacuum at 45 °C for 1 day to obtain the yellow crude extract.

The crude extracts were purified to DMY until recrystallizations, as reported by Liao et al. [50]. Briefly, 10 g of crude extract was soaked with 100 mL of ethanol for 60 min. Dried extracts were dissolved in 400 mL of water at 80 °C and crystallized at room temperature (25 °C) for 1 h. Recrystallization was repeated five times to obtain purified DMY on the basis of its different solubility in cold and boiled water.

The determination of DMY was carried out using high-performance liquid chromatography (HPLC) according to the method of He et al. [51]. This analysis was conducted on Agilent Series 1260 LC instrument (Agilent Technologies, Palo Alto, CA, USA) equipped with a Novapak C18 column (15.0 mm × 4.6 mm, 5 μm) and an ultraviolet spectrophotometric detector. Methanol-0.1% phosphoric acid solution (27:73, *w*/*w*) was used as the mobile phase with a flow rate of 1.0 mL/min. The column temperature was continually maintained at 25 °C, the sample injection volume was 10 μL, and the detecting wavelength was set as 294 nm.

The purity of DMY, as determined by three independent measurements, was about 94.5% ± 0.7%.

### 3.4. Antibacterial Activity

The antibacterial activity of DMY was tested using the disc-diffusion method established by Arima et al. [52]. Briefly, bacterial suspensions (1.5 × 10^8^ CFU/mL) cultured overnight in nutrient broth (NB) were spread on nutrient agar (NA) in Petri dishes. DMY solution, which was prepared by dissolving DMY in 75% ethanol, was dropped onto a paper disk (mm diameter, Xinhua No. 1 filter paper), and then this air-dried paper disk was placed on the agar. After incubating at 37 °C for 24 h, the diameter of the inhibition zone was measured with a transparent ruler and recorded in mm. The bacterial suspension and NB mixture without DMY solution was used as the negative control group.

For the tests of effect of calcium ions, CaCl_2_ was dissolved in DMY solution to get the concentrations of 0, 0.4, 0.8, 1.6, and 2.0 mg/mL.

For the tests of effect of thermal processing, DMY solution was heated at different temperatures (50, 70, and 100 °C) for different amount of time (10, 20, and 30 min).

For the tests of effect of different pHs, DMY solution was adjusted to pH 4.0, 5.0, 6.0, 7.0, 8.0, 9.0, and 10.0, respectively.

### 3.5. Determination of Minimum Inhibitory Concentration (MIC) and Minimum Bactericidal Concentration (MBC)

The MIC of DMY was determined using Duffy’s method with some modifications [53]. The inhibitory drug standard BH and the test substance DMY were dissolved in NB, respectively, to get concentrations of 20, 10, 5, 2.5, 1.25, 0.625, 0.3125, and 0.1562 mg/mL. Two milliliters of these solutions was mixed with 0.1 mL of 10^6^ CFU/mL bacterial suspension and cultured at 37 °C for 24 h.

MBC was determined by the method of Vaquero et al. [54]. Ten microliters of each sample suspension without bacterial growth in the MIC test was spread on NA and cultured at 37 °C for 24 h.

The MIC and MBC were interpreted as the lowest concentrations of DMY where it did not demonstrate visible growth of bacteria in NB and NA, respectively [55].

The NB without bacterial inhibitors was set as the negative control group.

### 3.6. Scanning Electron Microscopy (SEM) Observations

SEM assays were performed according to the method of Li et al. [37]. The *S. aureus* and *E. coli* suspensions were incubated with 0.625 and 0.3125 mg/mL DMY solution respectively, at 37 °C for 4, 14, and 24 h and then transported onto a cover slip. Slide-immobilized cells were fixed with 2.5% (*w*/*v*) glutaraldehyde in 0.1 mol/L phosphate buffer solution overnight at 4 °C and then washed with the same buffer. A graded ethanol series was used for cell dehydration. Then, the cells were observed by SEM (JSM-6701F, JEOL, Tokyo, Japan) after drying and gold coating. The images were generated using a 30 kV electron beam.

### 3.7. Determination of Extracellular Enzyme Activity

The activity of AKP, ALT, and AST was determined to evaluate the changes in the cell wall and membrane integrity according to the methods described by Cui et al. and Crowley [56,57]. Briefly, DMY was dissolved in bacterial suspension (10^6^ CFU/mL) to get the final concentrations of 1 × MIC and 2 × MIC, respectively. These mixtures were cultured at 37 °C and 150 rpm for different amounts of time and then centrifuged at 6000 rpm for 10 min. After being cultured for 0, 1, 2, 4, 6, and 8 h, the AKP activity in the supernatant was determined using the AKP kit and a UV/Vis spectrophotometer (Persee instrument Co., Ltd., Beijing, China) at 520 nm. After being cultured for 6, 12, 16, 20, 24, and 48 h, the activity of AST and AKP in the supernatant of 1 × MIC was determined using the AST and AKP kits respectively, at 340 nm. The bacterial suspension without DMY treatment was set as the negative control group.

### 3.8. Determination of the Bacterial Fluid Conductivity

The electrical conductivity was used to evaluate the permeability of cell membrane using the method described by Diao et al. [58]. DMY was added to the bacterial suspension (10^6^ CFU/mL) at final concentrations of 0.5 × MIC, 1 × MIC, and 2 × MIC, respectively. The bacterial suspension without DMY was set as the negative control group. The conductivity was measured at different time intervals using a conductivity meter (DDS-307, Jingmi Instruments Co., Ltd., Shanghai, China).

### 3.9. Determination of Oxidative Respiratory Metabolism

The initial solution was prepared by mixing 3.6 mL of phosphate buffer saline (PBS) (0.03 M, pH 7.3), 0.4 mL of glucose solution, and 1.0 mL of 10^6^ CFU/mL bacterial suspension in the tube. After being exposed to air for 5 min, the dissolved oxygen content in the inception stage was determined by a portable dissolved oxygen meter (JPBJ-608, Electric Scientific Instrument Co., Ltd., Shanghai, China) and marked as R_0_. The dissolved oxygen contents after adding the typical inhibitors (sodium phosphate tribasic dodecahydrate, iodoacetic acid, and malonic acid) and DMY were also measured, and this respiration rate was recorded as R_1_. In addition, after adding the mixture of DMY with different typical inhibitors, the dissolved oxygen content was further measured and recorded as R_2_. The inhibiting rate (I_R_) and superpose rate (S_R_) were calculated according to Equations (1) and (2), respectively [59].

(1) $$I_{R}=\frac{R_{0}-R_{1}}{R_{0}}\times 100\%$$

(2) $$S_{R}=\frac{R_{1}-R_{2}}{R_{1}}\times 100\%$$

### 3.10. Statistical Analysis

The results of all experiments are presented as the average values ± standard deviations of three identical experiments. To evaluate the integrity and permeability of the cell membrane, one-way ANOVAs and *t*-tests were performed at a significance level of *p* < 0.05.

## 4. Conclusions

During in vitro tests, DMY was proven to have ideal antibacterial activity against five tested food-borne bacteria. However, its antibacterial activity was shown to be very sensitive to pH, thermal processing, and metal ions. Furthermore, this study gave insight into its mode of action on *E. coli* and *S. aureus* as representatives of Gram-negative and Gram-positive bacteria. SEM suggested that DMY induces shriveling, aggregation, and adhesion of bacteria. The integrity of the cell membrane and wall was damaged, and thus intracellular ingredients, including AKP, AST, ALT, and electrolytes, leaked when treated with DMY. Besides, DMY can inhibit the respiratory metabolism of bacteria and mainly affect the TCA pathway. This research can broaden the application of DMY as a natural bacterial inhibitor with a broad antibacterial spectrum in the food industry.

## Figures and Tables

**Table 1:** The antibacterial ability of dihydromyricetin (DMY) against different bacteria.

Bacteria	DIZ (mm)				DMY (mg/mL)		BH (mg/mL)	
	Control	3.78 mg/mL	7.56 mg/mL	11.34 mg/mL	MIC	MBC	MIC	MBC
Gram positive								
*S. aureus*	ND	10.6 ± 0.5	15 ± 0.2	18 ± 0.3	0.625	2.5	0.625	2.5
*B. subtilis*	ND	8.1 ± 0.2	10.3 ± 0.4	12.3 ± 0.3	1.25	10	1.25	10
Gram negative								
*E. coli*	ND	9.8 ± 0.3	12.8 ± 0.3	15.5 ± 0.6	0.3125	2.5	0.625	5
*S. paratyphi*	ND	10.5 ± 0.3	11.2 ± 0.4	13.1 ± 0.5	0.625	2.5	1.25	5
*P. aeruginosa*	ND	10.1 ± 0.3	13.2 ± 0.3	16.3 ± 0.4	0.3125	2.5	0.3125	2.5

Values represent means of three independent replicates ± standard deviation (SD); ND represents not detected. BH: berberine hydrochloride; DIZ: diameter of the inhibition zone; MBC: minimum bactericidal concentration; MIC: minimum inhibitory concentration.

**Table 2:** The effect of DMY on the respiratory metabolism of bacteria.

Inhibitors	*S. aureus*		*E. coli*	
	I_R_/%	S_R_/%	I_R_/%	S_R_/%
DMY	20.62		19.24	
Malonic acid	28.26	11.47	24.18	9.62
Iodoacetic acid	7.47	17.16	8.26	15.47
Sodium phosphate tribasic dodecahydrate	19.18	24.96	17.26	19.38

I_R_ represents the inhibition rate of the representative inhibitor and DMY against bacteria.; S_R_ represents the superposition rate between DMY and the representative inhibitor.
